# Three Ways in Which Midline Regions Contribute to Self-Evaluation

**DOI:** 10.3389/fnhum.2013.00450

**Published:** 2013-08-02

**Authors:** Taru Flagan, Jennifer S. Beer

**Affiliations:** ^1^Department of Psychology, University of Texas at Austin, Austin, TX, USA

**Keywords:** self, optimistic bias, social cognition, frontal lobe, self-projection, motivation, person perception

## Abstract

An integration of existing research and newly conducted psychophysiological interaction (PPI) connectivity analyses suggest a new framework for understanding the contribution of midline regions to social cognition. Recent meta-analyses suggest that there are no midline regions that are exclusively associated with self-processing. Whereas medial prefrontal cortex (MPFC) is broadly modulated by self-processing, subdivisions within MPFC are differentially modulated by the evaluation of close others (ventral MPFC: BA 10/32) and the evaluation of other social targets (dorsal MPFC: BA 9/32). The role of DMPFC in social cognition may also be less uniquely social than previously thought; it may be better characterized as a region that indexes certainty about evaluation rather than previously considered social mechanisms (i.e., correction of self-projection). VMPFC, a region often described as an important mediator of socioemotional significance, may instead perform a more cognitive role by reflecting the type of information brought to bear on evaluations of people we know well. Furthermore, the new framework moves beyond MPFC and hypothesizes that two other midline regions, ventral anterior cingulate cortex (VACC: BA 25) and medial orbitofrontal cortex (MOFC: BA 11), aid motivational influences on social cognition. Despite the central role of motivation in psychological models of self-perception, neural models have largely ignored the topic. Positive connectivity between VACC and MOFC may mediate bottom-up sensitivity to information based on its potential for helping us evaluate ourselves or others the way we want. As connectivity becomes more positive with striatum and less positive with middle frontal gyrus (BA 9/44), MOFC mediates top-down motivational influences by adjusting the standards we bring to bear on evaluations of ourselves and other people.

## Introduction

**Figure 1:**
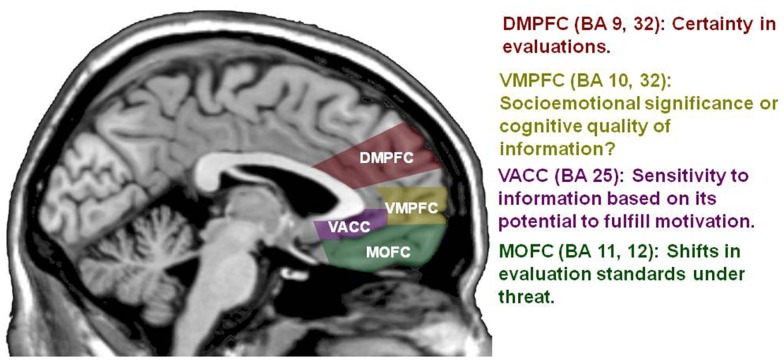
**A framework of cortical midline structures implicated in self-evaluation**. DMPFC, dorsomedial prefrontal cortex (BA 9, 32); VMPFC, ventromedial prefrontal cortex (BA 10, 32); VACC, ventral anterior cingulate cortex (BA 25); MOFC, medial orbitofrontal cortex (BA 11, 12).

The speculation that some midline regions contribute to self-processing stems from research conducted over a decade ago (Beer et al., 2006b). What have we learned since those initial studies found that medial prefrontal cortex (MPFC) is modulated by encoding and remembering information in relation to the self (e.g., Kelley et al., 2002; Macrae et al., 2004; Ochsner et al., 2005)? This article draws on existing research and newly conducted psychophysiological interaction (PPI) analyses to describe a new framework for the contribution of how a subportion of midline regions to social cognition (see Figure 1). The new framework builds on previous discussions by (a) positing a new role for the MPFC in social cognition and (b) moving past the MPFC to consider the importance of ventral anterior cingulate (VACC: BA 25) and medial orbitofrontal cortex (MOFC: BA 11) in aiding motivational influences on social cognition. Recent meta-analyses suggests that there are no midline regions that are exclusively associated with self-processing. For example, meta-analyses of studies of social evaluation (i.e., traits, personal abilities, etc.) find that the MPFC likely mediates psychological processes that are brought to bear on self-evaluation but also evaluations of other kinds of people (e.g., close others vs. non-close-others: Ochsner et al., 2005; Qin and Northoff, 2011; Murray et al., 2012; Roy et al., 2012). Whereas it was once thought that regions within VMPFC (BA 10/32) and DMPFC (BA 9/32) mediated person evaluation through the correction of self-projection, there are a number of issues that must be addressed before strong conclusions can be drawn. For example, current research provides more consistent evidence for the role DMPFC in certainty about self-evaluation even during tasks that require evaluations of other people. Furthermore, recent research suggests that VACC and MOFC are just as importantly involved in social cognition as the MPFC. Neural models of social cognition have not incorporated motivated processing which is a fundamental element of psychological models of the self (Beer, 2007). A growing body of research suggests that motivational influences on self- and other-evaluation are mediated by VACC and MOFC. VACC may mediate bottom-up sensitivity to information based on its potential for helping us evaluate ourselves or others the way we want (Beer, 2012a). MOFC may mediate top-down motivational influences on self-evaluation. Taken together, the new framework highlights the progress that has been made over the past decade: MPFC is involved in social cognition but does not mediate “self-specific” processes and two additional regions, VACC and MOFC, play an important role in motivated self- and other-evaluation.

## A New Conceptualization of the Role of MPFC in Social Cognition: Certainty in Evaluation?

While much has been learned about the role of MPFC in social cognition in the past decade, much more remains to be known. A series of studies in the early 2000s found that MPFC (BA 9/10/32) was modulated by both self-evaluation and evaluations of a political figure but modulation was greatest for self-evaluation (e.g., Kelley et al., 2002; Macrae et al., 2004; Ochsner et al., 2005). This research sparked interest in testing the possibility that functional MPFC subdivisions distinguished self-processing from the evaluation of other people. In contrast to this possibility, recent meta-analyses have shown that MPFC (BA 9/10/32) modulation is not exclusive to self; this region is modulated by both self-processing and processing about other people (Ochsner et al., 2005; Qin and Northoff, 2011; Murray et al., 2012; Roy et al., 2012). Instead of a self vs. other distinction, meta-analyses suggest a close other vs. non-close-other distinction. A ventral subdivision of this MPFC region (BA: 10/32, see Figure 1) is associated with self-processing and evaluations of close others (Ochsner et al., 2005; Qin and Northoff, 2011; Murray et al., 2012). A more dorsal subdivision (BA 9/32, see Figure 1) is associated with self-processing and evaluations of non-close-others (Ochsner et al., 2005; Qin and Northoff, 2011; Murray et al., 2012). Therefore, the next step toward understanding the contribution of MPFC to social cognition should be focused on understanding the psychological significance of MPFC’s broad association with self-processing in combination with the ventral to dorsal differentiation of processing about other people.

### DMPFC: Correcting self-projection or certainty in (social) judgment?

The correction of self-projection was one of the first psychological processes hypothesized to explain DMPFC’s association with both self-processing and processing of non-close-others. Psychological models suggest that one way we evaluate a new person is through the correction of self-projection, that is, by drawing on self-representation to the extent it is perceived as applicable to the new person (i.e., assumed similarity: Nickerson, 1999; Epley et al., 2004; Srivastava et al., 2010). Does DMPFC modulation reflect the corrective adjustment processes that are engaged to the extent that a new person is evaluated as dissimilar to the self? A different mechanism is suggested by an examination of the broader role of DMPFC in evaluation (including non-social evaluation) and psychological models of the interrelation between self-evaluation and evaluation of non-similar others. Specifically, DMPFC is associated with evaluation outside the social domain (Krain et al., 2006) and has been implicated in greater certainty about an evaluation (e.g., Krain et al., 2006; Bhanji et al., 2010). In contrast to the correction of self-projection hypothesis, it may be that certainty about self-evaluation explains why MPFC is modulated by the degree to which novel others are evaluated as different than the self.

#### Dissimilarity between the self and a novel person positively modulates DMPFC activation

Studies have consistently found that DMPFC (BA 9/32) activation parametrically increases to the extent that a novel person is evaluated as dissimilar to the self (Mitchell et al., 2005; Tamir and Mitchell, 2010). For example, these studies ask participants to report their own preferences (e.g., “how much do you look forward to going home for Thanksgiving?”) and to evaluate the preferences of strangers. The strangers are often manipulated to vary in their dissimilarity to the participant (e.g., have a different or similar political orientation, gender, or race). When neural activation is measured during the evaluation of strangers’ preferences, DMPFC (BA 9/32) is parametrically modulated by the dissimilarity between the participant’s own preferences and the preferences they assign to the strangers (Mitchell et al., 2005; Tamir and Mitchell, 2010). In other words, the more participants evaluate the strangers as dissimilar to themselves, the more DMPFC activation increases when participants are evaluating the stranger’s preferences.

The robust association between DMPFC modulation and dissimilarity between self and others has been theorized to reflect the role of DMPFC (BA 9/32) in correcting, that is, adjusting one’s own self-representation to estimate the experience of a stranger. This explanation stems from psychological models of person evaluation which suggest that people use themselves as a starting point and correct as needed to evaluate unknown others (Nickerson, 1999; Epley et al., 2004; Srivastava et al., 2010). So if you encounter someone who shares your political orientation and you have to evaluate their position on a particular issue, you are likely to use your own experience to evaluate the person’s position. However, if a new person does not share your political orientation, then you cannot simply use your own experience and your evaluation will likely correct for the extent to which the person differs in political orientation (e.g., a liberal may feel that a self-representation might partially apply to a stranger who is a moderate but not apply at all to someone who is conservative).

Does MPFC modulation reflect a correction of self-projection in social evaluation or is there another explanation that warrants examination before a strong conclusion can be drawn? There are a number of findings which raise the possibility that DMPFC modulation may instead reflect greater certainty in evaluation rather than correction of self-projection. For example, the previous studies have looked at DMPFC modulation during the evaluation of others. How does this compare to DMPFC modulation during self-evaluation and is it consistent with a correction of self-projection explanation? Meta-analyses find that DMPFC (DMPFC is a label that is used in various ways in previous literature; the present article draws on published meta-analyses and uses the term DMPFC as label for relevant portions of BA 9/32. Within BA 9, *z* ranges from 20 to 42 in MNI coordinates, see Figure 1) is activated by both self-processing and evaluations of other people not personally known by the participant. In direction comparisons, some meta-analyses find that this activation is relatively greater for unknown others while other meta-analyses find no difference in this region’s activation for self-processing and evaluation of unknown others (Self vs. Other comparison: Ochsner et al., 2005; Qin and Northoff, 2011; Roy et al., 2012). If the DMPFC reflects a correction of self-projection that occurs while evaluating another person, then it is puzzling why DMPFC is modulated by self-evaluation to the same degree as the evaluation of a person who is not personally known but assumed to be dissimilar to the self. If this region of DMPFC indexes correction away from a self-representation, why would this correction be engaged when evaluating oneself?

#### An alternate conceptualization of why DMPFC is modulated by self-other dissimilarity: certainty about the evaluation

Although it has not received much empirical attention, there is an alternate mechanism which could explain the pattern of DMPFC modulation found in these studies of self-evaluation and evaluation of strangers. Research on evaluation in non-social domains finds an association between DMPFC activation and greater certainty in evaluation (e.g., Krain et al., 2006; Bhanji et al., 2010; Eldaief et al., 2012). An integration of the psychological research on the interplay between self- and other-evaluation with the established association between DMPFC and evaluation certainty suggests that the DMPFC modulation found in paradigms involving self-evaluation and evaluation of strangers is tracking certainty in self-evaluation.

It has already been shown that certainty about one’s self-evaluation modulates DMPFC activation (D’Argembeau et al., 2012). Studies on self-referent processing ask participants to rate the self-descriptiveness of personality traits and find that MPFC activation (extending into the DMPFC) is increased to the extent the traits are evaluated as self-descriptive (e.g., Moran et al., 2006; D’Argembeau et al., 2012). A personality trait may be evaluated as self-descriptive because people are certain about their association with the trait or they may be motivated to see themselves as characterized by that trait. One study delved further into these underlying reasons and found that a region within DMPFC was modulated by degree of certainty that the trait applied to self (D’Argembeau et al., 2012).

DMPFC is associated with certainty about evaluation both for non-social tasks and self-evaluation; but how can an increase in certainty explain why DMPFC activation increases to the extent we evaluate people who are dissimilar to the self? Wouldn’t we be feeling uncertain when evaluating people we presume do not share our own qualities? Psychological research finds that evaluations of dissimilar others elicits a spontaneous self-evaluation and ironically solidifies our certainty about our own opinions and attitudes. In fact, certainty about our own preferences increases to the extent that the we perceive the target of our evaluation to be dissimilar to ourselves (Holtz and Miller, 2001; Holtz and Nihiser, 2008). In other words, this research suggests that rather than using the self as an anchor for evaluating other people (i.e., self-projection), the evaluation of other people triggers a spontaneous self-evaluation. And the more we evaluate someone to be different from us, the more we feel certain about where we stand on that attribute. Therefore, the increasing DMPFC activation found during a task that requires the evaluations of others could also be indexing an aspect of concomitant, spontaneous self-evaluations. Specifically, DMPFC activation may be modulated by increased certainty about the self to the extent that the target of evaluation is perceived as dissimilar to the self.

#### Implications of an association between DMPFC and certainty about self-evaluation

If DMPFC modulation does reflect certainty in self-evaluation, then a reconceptualization of self-evaluation localizer tasks may be warranted. Some studies have used a self-referent processing task (i.e., asking participants to rate their own personality traits compared to rating the personality traits of a political figure) as a way of localizing neural regions associated with self-processing for subsequent tasks. Future research is needed to understand whether this task identifies regions within DMPFC that index the intended rich psychological aspects of self or simply certainty in evaluation (i.e., on average, we are likely more certain about self-evaluation than evaluation of a political figure only seen in the news).

### VMPFC: Socioemotional connection or firsthand experience?

The correction of self-projection or certainty might explain the contribution of DMPFC to social cognition but what about the more ventral subdivision of MPFC (VMPFC) that is associated with evaluations of self and intimate others (VMPFC is a label that is used in various ways in previous literature; the present article draws on published meta-analyses and uses the term VMPFC as label for relevant portions of BA 10/32; *z* range −2 to 8, see Figure 1, Ochsner et al., 2005; Qin and Northoff, 2011; Murray et al., 2012; Roy et al., 2012)? Two different hypotheses have been proposed: the correction of self-projection (Mitchell et al., 2005; Tamir and Mitchell, 2010) and self-relatedness (Northoff et al., 2006; Krienen et al., 2010; Murray et al., 2012; Roy et al., 2012). Currently, there is only mixed support for the correction of self-projection perspective. The hypothesis that VMPFC may mediate self-relatedness is more consistent with the available data but more research is needed to unpack the psychological meaning of self-relatedness.

#### VMPFC modulation and the correction of self-projection? current studies find inconsistent associations

Unlike the DMPFC, VMPFC modulation has not shown a consistent pattern of association with evaluations of others as function of self-other dissimilarity (Mitchell et al., 2005; Krienen et al., 2010; Tamir and Mitchell, 2010). For example, an initial study asked participants to rate the preferences of unknown others (i.e., pleasure at having their photograph taken: Mitchell et al., 2005). VMPFC activation during the preference-evaluation task decreased to the extent that the unknown others were evaluated as dissimilar to the self in a post-scan procedure. Yet a follow-up analysis found a different pattern: VMPFC activation did not show a parametric association and it showed a positive (i.e., opposite) association to dissimilarity. VMPFC showed little change (in relation to baseline) during evaluations of other people who were evaluated (to any degree) as dissimilar from the self and a significant deactivation (in relation to baseline) when evaluating similar others (Tamir and Mitchell, 2011). It has been suggested that the different findings might indicate the existence of different neural mediation for computing global vs. specific dissimilarity. Dissimilarity was operationalized as a person’s global political affiliation on the one hand (Mitchell et al., 2006) and trial-by-trial specific preferences on the other (i.e., Tamir and Mitchell, 2010). However, another series of studies found no association at all between VMPFC (BA 10) modulation and similarity between close others or strangers (Krienen et al., 2010). This research instead found that VMPFC shows increased activation for self-evaluations and evaluations of close others (regardless of similarity) and less activation for unknown others. Even if future research were to flesh out a robust association between VMPFC modulation and evaluations of dissimilar others, the correction of self-projection explanation still suffers from a parallel set of problems mentioned above in relation to DMPFC. Meta-analyses find that VMPFC activation is modulated by both self-evaluation and evaluation of intimate others (Ochsner et al., 2005; Qin and Northoff, 2011; Murray et al., 2012). It is unclear why people would need to correct the use of their self-representation when evaluating themselves or why they would use a self-projection process to evaluate someone they know well.

#### VMPFC modulation and self-relatedness of social evaluation: a socioemotional or cognitive mechanism?

Another predominant hypothesis arising from current social evaluation research is that VMPFC marks “self-relatedness,” that is, the socioemotional connection between the self and the person being evaluated (Northoff et al., 2006; Krienen et al., 2010; Murray et al., 2012; Roy et al., 2012). Self-relatedness is a socioemotional variable reflecting the extent to which the evaluation process draws on affectively rich, self-representations. Psychological models of social evaluation suggest that self-representations may be activated by evaluations of close others but not for the same reason as for unknown others. “Close others” are often defined by the extent to which representations of those people are associated with self-representations (Aron et al., 1992). It is not the case that the self-representation is theorized to serve as a starting point for evaluating the close other (i.e., a self-projection-like process which is then subject to correction). Instead, the evaluation of a close other draws on a representation of the close other that is emotionally charged because of its association with the self-representation. From this perspective, VMPFC is modulated by self-evaluation and evaluation of close others because those evaluations have a unique affective or socioemotional significance.

However, it may not be that VMPFC marks whether social evaluations are “self-like” in a socioemotional sense. In the existing research, socioemotional relation between the self and another person has always been confounded with the quality of information (e.g., cognitive representation) used to make an evaluation. We simply have a different class of information to draw on when we evaluate ourselves and people we actually know (e.g., greater complexity, abstraction, actual experience) compared to unknown others. A novel person and a romantic partner elicit different emotional reactions but they also elicit different cognitive representations. For both the self and romantic partners, there is a long history of storing person information which creates a more elaborated representation that includes both abstract and biographical information when compared to representations that could be used to evaluate someone who is relatively unknown (e.g., Sherman and Klein, 1994; Kihlstrom et al., 2003). A brain region that indexes one or more cognitive qualities that are emphasized in the representations of people we know well (i.e., self, close other) would also behave like the VMPFC across these social evaluation tasks as reviewed above (i.e., similar modulation across self-evaluation and evaluation of close others but less modulation for unknown others: Ochsner et al., 2005; Krienen et al., 2010; Qin and Northoff, 2011; Murray et al., 2012; Roy et al., 2012). This raises the possibility that the contribution of VMPFC to social cognition is more a cognitive (rather than affective) “self-relatedness.” From this perspective, VMPFC may mediate a quality of the kind of information that feeds into self-evaluations that is also available for evaluations of people we actually know (but not as much for unknown others).

## Expanding Beyond MPFC (BA 9/10/32): VACC (BA 25) and MOFC (BA 11) Mediate Motivational Influences on Bottom-Up and Top-Down Processing of Social Targets

Despite the heavy focus on MPFC (BA 9/10/32), an emerging body of literature suggests that at least two other midline regions are just as important for social cognitive processing: VACC (BA 25) and MOFC (BA 11) (see Figure 1). VACC and MOFC mediate motivational aspects of self-processing. Motivation has been ascribed a central role in psychological models of self-processing (Kunda, 1990; Robins and John, 1997). For example, self-evaluations tend to be positively tinged (also described as “self-serving,” “the above average effect,” “self-flattering,” “self-enhanced” “optimistic bias”: Alicke, 1985; Taylor and Brown, 1988; Dunning et al., 1989; Chambers and Windschitl, 2004). Self-evaluations are described as positively tinged to the extent that they are more positive than warranted by some other criterion and this positive slant may even be pre-potent, that is, the default mode of self-evaluation (Beer, 2007). Cognitive load makes self-evaluation even more positively tinged (Paulhus et al., 1989; Kruger, 1999; Koole and Dijksterhuis, 2001; Lench and Ditto, 2008; Beer and Hughes, 2010; Beer et al., 2013). Furthermore, the positive tinge of self-evaluation is not circumscribed to the lab (Dunning et al., 2004). People will wager money that their positively tinged views are accurate (Williams and Gilovich, 2008), expect that other people will share their positively tinged views (Hepper et al., 2011), and experience different life trajectories based on the extent of their positive slant (Robins and Beer, 2001). A positive tinge also pervades evaluations of close others but is less evident in evaluations of unknown others (Suls et al., 2002). A positive tinge may arise because people use incomplete information when making a social evaluation (e.g., using the first thing that comes to mind which happens to be positive: Chambers and Windschitl, 2004). However, a positive tinge can also arise from the motivation to cast oneself or a close other in a positive light (i.e., self-flattery: Taylor and Brown, 1988; Sedikides and Gregg, 2008). Despite the central role of motivation in psychological models of self- and person evaluation, neural models of self-processing have paid little attention to motivation (Beer, 2007, 2012a). Recent research that addresses this gap suggests that (a) VACC may be modulated by opportunities that have the potential to accomplish a motivated self-evaluation (i.e., motivational influences on bottom-up processing) and (b) MOFC may be modulated by the extent to which the motivation to cast oneself in a positive light requires the adjustment of evaluation thresholds across contexts (i.e., top-down processing).

### VACC: Motivational influences on bottom-up processing

VACC may mediate bottom-up sensitivity to opportunities that have the potential to affirm the way someone wants to evaluate themselves; however, it does not predict whether the opportunity will successfully lead to motivated self-evaluation (Moran et al., 2006; Sharot et al., 2007; Beer and Hughes, 2010; Hughes and Beer, 2012a). Social psychological theories of self- and other-evaluation often characterize these evaluations in terms of the contribution of bottom-up and top-down processes (e.g., Duncan, 1976; Shavelson and Bolus, 1982; Devine, 1989; Fiske and Neuberg, 1990; Brown et al., 2001; Beer, 2012b). “Top-down” and “bottom-up” are terms that are used widely, but somewhat differently across fields. In the case of motivated self-evaluation, these terms can be used to distinguish between subjective and objective construals of information. People may be motivated to see themselves in a particular way and, therefore, interpret information in a top-down, subjective manner that ensures the information can be used to accomplish a motivated self-evaluation. Or the motivation may affect the kind of information that is distinguished from other kinds of information (i.e., the influence of the motivation on relatively bottom-up processing: e.g., Brown et al., 2001).

The influence of motivation on relatively bottom-up processing of information can be illustrated by the example of people filling out an online dating profile who want to portray themselves as especially athletic compared to other people. If people scan the activities checklist with the goal of portraying themselves as especially athletic, then we predict that VACC will be modulated by activities on the checklist that objectively involve sports vs. activities that reflect poorly on athleticism (e.g., watching television). Similarly, someone with the goal of portraying themselves as artistic would show greater VACC activation when reading checklist activities that objectively involve artistic pursuits vs. all of the other options. In this way, VACC activation is implicated in the influence of motivation on bottom-up processing of the checklist because VACC modulation distinguishes between opportunities that are objectively consistent vs. inconsistent with the activated motivation. VACC is not implicated in purely top-down processing because research suggests that it would not predict the extent to which someone claims to be especially involved in each sport compared to other people (i.e., the success of the top-down goal of portraying oneself as particularly athletic). In other words, VACC modulation does not predict the extent to which the meaning or interpretation of the checklist activities are subjectively construed to fit with the activated motivation nor does it predict reported self-evaluation on those checklist activities. Instead, we hypothesize that VACC is modulated by a preliminary and relatively more bottom-up step of motivated evaluation: delineating the existence of opportunities that objectively have the potential to cast yourself in particular light.

#### VACC activation differentiates positive valence from negative valence, especially for social targets we want to see in a positive light

The distinction between opportunity for motivated evaluation and success in motivated evaluation is important because they have been conflated in the current literature (Beer, 2007, 2012a). It is inappropriate to use the term “bias” (e.g., positively tinged) to label a self-endorsement of a positive trait or likelihood of a positive future event. There is no way to know whether someone has successfully achieved a positively tinged evaluation simply because they are rating positive traits or future events as particularly self-descriptive. The person may truly possess high levels of that trait and be predisposed to a positive future or they may not. A response can be characterized as “biased” or positively tinged (rather than merely positive) when it is more positive than warranted by a benchmark criterion (Beer, 2007, 2012b).

For example, one way that positively tinged self-evaluation has been operationalized is the extent to which people inflate their own standing when comparing themselves to other people (Taylor and Brown, 1988; Chambers and Windschitl, 2004). This line of research often asks participants to make social-comparative judgments. That is, participants are asked to evaluate how much they possess personality traits in comparison to their average peer (i.e., much less, about the same or much more than someone of the same, age, community, education level, etc.). When participants’ social-comparison evaluations are averaged across hundreds of personality traits, their average evaluation, by definition, should be somewhere near the average peer benchmark. However, the majority of people report having significantly higher levels of positive personality traits and significantly lower levels of negative traits than their average peer (Taylor and Brown, 1988; Chambers and Windschitl, 2004). In this social-comparison task, VACC is modulated by the condition that includes positive personality traits (compared to negative personality traits) but it does not predict the extent to which someone reports an overall significantly more desirable personality in comparison to their average peer (Beer and Hughes, 2010; Hughes and Beer, 2012a).

VACC has been implicated in the detection of emotionally significant, that is, valenced information in a variety of tasks (compared to non-valenced information: Bush et al., 2000). However, research on social cognition has shown that VACC modulation may differentiate between particular classes of valence depending on motivational state. When people evaluate well-liked social targets (e.g., the self, romantic partner, well-liked roommate), VACC activation differentiates trials where endorsement would portray the target in a positive light (i.e., desirable personality traits, likelihood of a positive future) from trials where endorsement would portray the target in a negative light (i.e., undesirable personality traits, likelihood of a negative future: Moran et al., 2006; Sharot et al., 2007; Beer and Hughes, 2010; Hughes and Beer, 2012a). However, when there is reduced motivation to portray the target in a positive light (i.e., personality traits that are not considered central to one’s self-view: Sedikides and Gregg, 2008; a non-close other: Suls et al., 2002), VACC activation is less likely to differentiate trials on the basis of how endorsement would portray the target (i.e., the self: Moran et al., 2006; an assigned college roommate: Hughes and Beer, 2012a). This research suggests that VACC is important for identifying opportunities to portray someone in a particular light but it does not predict whether the opportunity actually leads to successful motivated evaluation.

#### Bottom-up sensitivity to information based on its potential to affirm motivated self-evaluations: connectivity between VACC and MOFC

**Figure 2:**
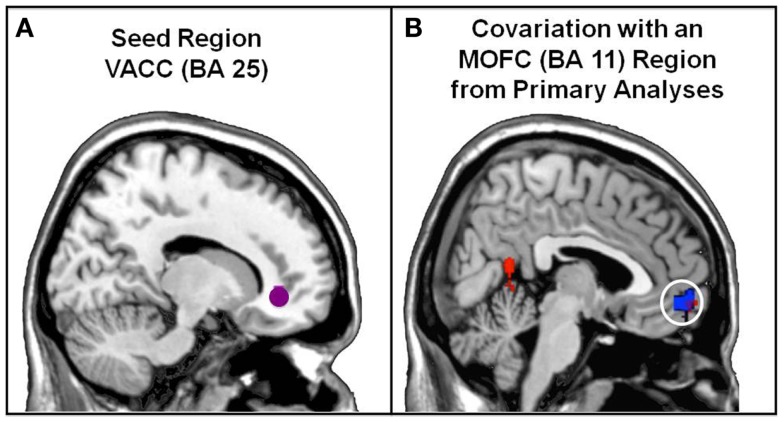
**PPI connectivity analyses for the VACC seed associated with social comparisons about Positive (i.e., desirable) vs. Negative (i.e., undesirable) personality traits**. **(A)** Each participant’s time series was extracted from the VACC seed (5 mm radius sphere around group peak: 14, 38, −4 from the Positive vs. Negative contrast, Beer and Hughes, 2010). **(B)** The VACC seed shows relatively more positive covariation with an MOFC region. This MOFC region overlaps with the MOFC region that regulates the extent to which social comparisons are positively tinged (red: MOFC region found in PPI analyses; blue: MOFC region found in Beer and Hughes, 2010; purple: overlap between MOFC region in connectivity and primary analyses).

Psychophysiological interaction connectivity analyses (Friston et al., 1997) conducted on previously published results (Beer and Hughes, 2010) further supports the hypothesis that VACC (BA 25) mediates a preliminary step but not the ultimate success of motivated evaluation (for the full set of results, see Figure 2; Table 1).

**Table 1:** **PPI connectivity analyses with VACC seed from data published in Beer and Hughes (2010)**.

	Side	Region of activation	BA	*x*	*y*	*z*	*z*-stat	No. of voxels
**POSITIVE > NEGATIVE**								
Increased positive covariation	L	Lingual gyrus	18	−8	−48	−4	4.20	1463
	L	Postcentral gyrus	48	−52	−18	32	3.72	305
	L	Cerebellum	18	−26	−74	−20	3.33	212
	R	Medial orbitofrontal cortex	11	8	52	−10	3.40	123
	R	Supramarginal gyrus	48	56	−18	26	2.82	24
	R	Cerebellum	20	46	−38	−32	2.95	22
	R	Postcentral gyrus	2	46	−38	62	2.95	21
	R	Midcingulate	24	4	16	34	2.76	19
	R	Cerebellum	19	30	−78	−18	2.84	16
	L	Posterior cingulate	23	0	−22	40	2.76	15
Decreased positive covariation	R	Middle temporal gyrus	21	48	−48	12	3.53	1051
	L	Inferior temporal gyrus	20	−42	−18	−20	3.52	677
	R	Inferior temporal gyrus	20	62	−16	−24	4.03	427
	R	Inferior frontal gyrus	40, 11	26	28	−20	3.38	301
	L	Inferior temporal gyrus	37	−50	−52	−12	3.20	231
	L	Middle temporal gyrus	41	−44	−48	22	3.27	225
	L	Inferior parietal lobule	40	−54	−46	44	3.49	161
	R	Temporal pole	20	46	6	−36	3.07	113
	R	Fusiform	20	28	−6	−46	3.32	67
	R	Inferior temporal gyrus	20	70	−36	−20	3.08	65
	L	Middle temporal gyrus	21	−60	−36	0	3.08	64
	R	Parahippocampal gyrus	28	20	−4	−28	2.96	54
	R	Inferior temporal gyrus	36	38	−2	−28	2.86	49
	L	Superior frontal gyrus	8	−18	26	54	3.02	28
	R	Inferior frontal gyrus	48	36	24	12	3.00	23
	R	Middle temporal gyrus	39	52	−74	14	2.91	22
	R	Inferior frontal gyrus	45	62	30	12	2.91	15
	L	Putamen		−26	8	4	2.71	15
	L	Insula	48	−30	12	12	2.88	12

##### Methods

Whole-brain PPI analyses were conducted in order to investigate the functional connectivity of the VACC region that differentiated social-comparative evaluations made in the Positive condition from the social-comparative evaluations made in the Negative condition (Beer and Hughes, 2010). Specifically, participants rated how much they had desirable (Positive condition) and undesirable (Negative condition) personality traits in comparison to their average peer. Imaging data were preprocessed using the FSL software toolbox [Oxford Center for Functional Magnetic Resonance Imaging (FMRIB); Smith et al., 2004]. Functional images were motion corrected using MCFLRT (Jenkinson et al., 2002) and non-brain structures were stripped from functional and structural volumes using the Brain Extraction Tool (BET; Smith, 2002). Images were then smoothed (8 mm full-width half-maximum) and normalized to MNI-152 space during preprocessing. Parameters for normalization into a standard space were obtained by multiplying the transformation matrices across a two-step process in which the functional images were registered to the MP-RAGE (6 DOF affine transformation), and the MP-RAGE was registered to the MNI-152 template (12 DOF affine transformation).

Functional Magnetic Resonance Imaging analysis was performed using FSL’s FEAT (FMRI Expert Analysis Tool version 5.98). A fixed-effects analysis modeled event related responses for each participant. Responses made in the Positive and Negative conditions were modeled as events using a canonical hemodynamic response function with a temporal derivative. Motion regressors were modeled as regressors of no interest. Each participant’s time series was extracted from the VACC seed found in the group analyses of the Positive vs. Negative condition (5 mm radius sphere around group peak: 14, 38, −4 from the Positive vs. Negative contrast, Beer and Hughes, 2010). Two PPI regressors were created: the interaction of the time series of the VACC seed with (a) the time series of the Positive condition regressor and (b) the time series of the Negative condition regressor.

A subsequent fixed-effects analysis was conducted modeling the following regressors: (a) Positive condition regressor, (b) Negative condition regressor, (c) temporally filtered activity across the time course from the VACC seed region, (d) PPI regressor for the Positive condition, and (e) PPI regressor for the negative condition. The PPI regressors were contrasted in a GLM. A second-level analysis created contrast estimates for each participant by collapsing across the two runs, treating runs as a fixed effect. FEAT’s FLAME module (FMRIB’s Local Analysis of Mixed Effects; Smith et al., 2004) was used to preformed mixed effects analysis which created group average maps for contrasts of interest (*p* < 0.005, uncorrected). The significance threshold was chosen because it is the recommended threshold for striking an optimal balance between Type I and Type II error when reporting analyses of brain activation in relation to complex psychological processes; simulation studies show that other significance thresholds raise the possibility of Type II error beyond acceptable limits (Lieberman and Cunningham, 2009). As the first report of functional connectivity in relation to motivated self-evaluation, the goal was to be as inclusive as reasonably possible to avoid missing true effects.

##### Results

When people make social comparisons about desirable traits, VACC shows relatively more positive covariation with a portion of MOFC (BA 11) that was found to regulate the extent to which social-comparative evaluations are positively tinged in the primary analyses. Although directionality cannot be determined from PPI analyses, it is possible that VACC is involved in analyzing the opportunities afforded by the content of an evaluation (i.e., a desirable trait vs. an undesirable trait). That information may then be processed upstream by the MOFC before an evaluation is expressed.

### MOFC: Motivational influences on top-down processing

As mentioned above, the MOFC is implicated in self-evaluation. How should we conceptualize its role? Take the example mentioned earlier: people who view themselves as particularly athletic complete an activity checklist on an online dating profile. Their expectation may be met when they are able to endorse participation in numerous sports on the checklist. But if they find themselves able to only endorse involvement in just one or two of the numerous sports possibilities, they may have one of two possible reactions. If their self-esteem is not staked on their athletic ability, they might realize that they are not so different from other people in this regard. However, if the procedure threatens their self-esteem, they may react defensively by changing their evaluation threshold in such a way that they can evaluate themselves as having even more superior athleticism. For example, they may evaluate degree of athleticism based on the intensity of involvement in a particular sport, rather than on the number of sports activities they can endorse on the checklist. In one case, the initial expectation of portraying oneself as athletic is dismissed during activity endorsement (i.e., an initial top-down influence is controlled). In the case where self-esteem is threatened, motivation to portray oneself in a particular way exhibits a top-down influence on activity endorsement by biasing the standards with which the evaluation is made. MOFC modulation has been associated with both of the examples above: realizing the self is not as special as expected and defensive reactions when self-esteem is threatened. Connectivity analyses suggest that MOFC modulation likely reflects different psychological processes across these circumstances. In particular, a network involving MOFC and (a) relatively more positive covariation with striatum and (b) relatively less positive covariation with middle frontal gyrus may aid self-evaluations that protect the self in the face of self-esteem threat. However, MOFC activation found in association with dismissing the influence of a self-evaluation motivation does not show such connectivity. In this way, MOFC may mediate top-down motivational influences on social evaluation by supporting changes in evaluation standards to either facilitate or control an activated motivational state.

#### When self-esteem is not at stake: OFC function is negatively associated with positively tinged social evaluations

Both neuroimaging and lesion studies have shown that reduced MOFC function is associated with positively tinged social evaluations (i.e., self and close others: Beer et al., 2003; Beer et al., 2006a, 2010; Beer and Hughes, 2010; Hughes and Beer, 2012a). This relation holds across various operationalizations of self-flattery: the difference in the way you see yourself compared to how others view you, self-evaluation of task achievement compared to actual task performance on an unimportant task, and base rates of social comparisons.

A series of studies found that patients with OFC damage tend to view their social behavior in a positively tinged manner (Beer et al., 2003, 2006a). In one study, patients with OFC damage were socially disinhibited compared to healthy control participants yet they expressed greater pride in their social behavior (i.e., inappropriate teasing of strangers: Beer et al., 2003). Another study found that patients with OFC damage did not evaluate the appropriateness of their social behavior any differently than healthy control participants or participants with dorsolateral prefrontal cortex (DLPFC) damage. Yet outside observers, blind to participant status, rated the social behavior of the patients with OFC damage to be significantly more inappropriate than the other groups (i.e., too familiar for an interaction with a stranger: Beer et al., 2006a).

Neuroimaging results complement the lesion studies: reduced OFC activation (BA 11) is associated with positively tinged evaluations of one’s task performance and personality (Beer and Hughes, 2010; Beer et al., 2010; Hughes and Beer, 2012a). In one study, participants estimated their confidence in their answers to a trivia task. Reduced OFC activation (BA 11) predicted the extent to which participants were overconfident about their incorrect trivia answers (Beer et al., 2010). Reduced OFC activation also predicts the extent to which people view themselves and their romantic partners to have significantly more desirable personalities than their peers. As mentioned above in the section on VACC function, these studies ask participants to compare themselves or their romantic partners to an average peer (i.e., a person who is the same gender, age, from the same community, university campus, etc.). When these social-comparative judgments of personality traits are averaged across hundreds of traits, each participant (or their romantic partner) should, by definition, be evaluated as comparable to their average peer. Whereas VACC activation showed no relation, reduced OFC activation is associated with the extent to which people evaluate themselves (Beer and Hughes, 2010) or their romantic partners (Hughes and Beer, 2012a) to have significantly more positive traits and significantly fewer negative traits than their average peer. Taken together, these studies provide robust evidence that reduced OFC activation predicts positively tinged evaluations on a trial-by-trial, condition, and individual difference basis.

#### The case of self-esteem defense: a positive association between OFC activation and self-protection

There is an exception to the findings described above: increased MOFC (BA 11) activation predicts self-evaluations in situations where self-esteem comes under attack (Hughes and Beer, 2013). Self-esteem is typically threatened when people receive negative feedback about their personality, academic abilities, or skills (Baumeister et al., 1993; Leary et al., 1998; vanDellen et al., 2011). People cope with self-esteem threat by inflating the positively tinged nature of their self-evaluation (including social comparisons: Beer et al., 2013 and see vanDellen et al., 2011 for review). The lesion and fMRI research reviewed above did not include any manipulations to threaten self-esteem. What happens to the underlying neural modulation when social-comparison judgments are used to cope with self-esteem attack? In other words, what neural regions mediate self-evaluations that are self-flattering (e.g., positively tinged with the purpose of protecting the self against a self-esteem threat)? One fMRI study addressed this question by using the very same social-comparison evaluation as a previous study (Beer and Hughes, 2010) but added in a self-esteem threat manipulation (Hughes and Beer, 2013). Participants learned that other students had found them either likable or unlikable and then evaluated how their personalities compared to their peers. Consistent with previous research, evaluations made after learning that others found them unlikable were even more self-flattering (compared to learning that others found them likable). The extent to which social comparisons became even more self-flattering as a function of self-esteem attack was positively associated with increased MOFC modulation (Hughes and Beer, 2013). Therefore, this study found that MOFC modulation predicted a change in self-evaluation but, in the case of self-esteem attack, it shows a positive association with self-protection.

Although the studies on social comparison (Beer and Hughes, 2010; Hughes and Beer, 2013) provided a rigorous test of the association between MOFC modulation and self-evaluation as a function of self-esteem threat, they were not designed to pinpoint the underlying psychological process that explained the association. One study has begun to address this issue by using Signal Detection Theory to investigate the neural associations of self-evaluations used to protect one’s self-esteem (Hughes and Beer, 2012b). Just as people tend to inflate their social standing on personality traits, they tend to claim knowledge about concepts beyond what they actually know or could know (i.e., overclaim knowledge: Paulhus et al., 2003; Beer et al., 2010). However, when self-esteem is potentially at stake (i.e., their false claims could be discovered), people reduce the extent to which they overclaim knowledge (Paulhus et al., 2003) or inflate their social standing on personality traits (McKenna and Myers, 1997). In conditions where false claims would make them look foolish, people protect their self-esteem by adopting a different standard (i.e., decision threshold) for claiming knowledge which consequently reduces overclaiming. An fMRI study found that MOFC (BA 11) modulation was positively associated with the shift toward a more conservative standard in conditions where participants would look foolish if they were to make false claims of knowledge (i.e., they were warned that some concepts in the list did not exist: Hughes and Beer, 2012b).

#### A top-down role of MOFC in social evaluation

Consistent with the hypothesis that self-evaluations used to cope with self-esteem threat are distinct from self-evaluations made in the absence of threat, a relatively consistent pattern of functional connectivity emerged in the studies that investigated the impact of self-esteem threat on self-evaluations (Hughes and Beer, 2012b, 2013) and was distinct from the pattern found in a parallel social-comparison procedure that did not manipulate self-esteem threat (Beer and Hughes, 2010, see Table 2).

**Table 2:** PPI connectivity analyses with MOFC seed*.

	Side	Region of activation	BA	Coordinates			*z*-stat	No. of voxels
				*x*	*y*	*z*		
**ACCOUNTABLE (i.e., THREAT) > UNACCOUNTABLE (Hughes and Beer, 2012b)**								
Increased positive covariation	R	Putamen		22	−10	6	2.75	13
Reduced positive covariation	L	Middle frontal gyrus	9	−24	52	30	2.82	12
**THREAT > NO THREAT (Hughes and Beer, 2013)**								
Increased positive covariation	R	Caudate		14	16	−10	2.75	22
Reduced positive covariation	L	Middle frontal gyrus	9	−22	30	46	2.76	21
	L	Middle frontal gyrus	44	−40	24	38	2.68	13
	L	Middle frontal gyrus	9	−30	32	40	2.61	9
**SPECIFIC > BROAD (TRAIT BREADTH, THREAT NOT INCLUDED: Beer and Hughes, 2010)**								
Reduced positive covariation	L	Thalamus		−14	−16	−4	3.18	112
	L	Superior frontal gyrus	10	−10	56	12	3.34	55
	L	Cerebellum		−2	−60	−32	2.88	31
	L	Supramarginal gyrus	2	−58	−26	40	2.81	30
	R	Fusiform gyrus	20	36	−14	−30	3.08	20
	L	Superior temporal pole	38	−36	26	−30	2.98	18
	L	Superior temporal gyrus	38	−56	4	−10	2.79	14
	R	Fusiform	37	34	−66	−10	2.78	14
	L	Parahippocampal gyrus	37	−26	−36	−10	2.85	13
	L	Pallidum		−24	−10	−4	2.77	10
	L	Fusiform	36	−32	0	−40	2.67	10

*No regions found for increased positive covariation in Beer and Hughes (2010).

##### Methods

Whole-brain PPI analyses were conducted in order to investigate the functional connectivity of MOFC during social comparisons in the presence and absence of self-esteem threat from three previously published datasets (Dataset 1: Hughes and Beer, 2013; Dataset 2: Hughes and Beer, 2012a; Dataset 3: Beer and Hughes, 2010). For all three datasets, the preprocessing steps were the same as described earlier for the PPI analyses of the VACC seed. PPI analyses were conducted as follows. In Dataset 1 (Hughes and Beer, 2013), participants made social-comparative evaluations of their personality traits while the presence of self-esteem threat was manipulated. In other words, participants evaluated how their personality traits compared to an average peer after just learning that a majority of peers found them unlikable (Threat condition) or a majority of peers found them likable (No Threat condition). As previously published, increased MOFC activity is associated with positively tinged evaluations of one’s personality in the Self-esteem Threat condition (both a main effect and individual differences in evaluations made in the Threat vs. No Threat condition: Hughes and Beer, 2013). Each participant’s time series was extracted from the MOFC seed (group peak: −12, 54, −14 from the Threat vs. No Threat contrast, Hughes and Beer, 2012a). Two PPI regressors were created: interaction of the time series of the MOFC seed with (i) the time series of the Threat condition regressor and (ii) the time series of the No Threat condition regressor.

In Dataset 2 (Hughes and Beer, 2012b), participants evaluated their familiarity with blocks of information they believed would make them appear intelligent while their awareness of the exposure of fake claims was manipulated. Specifically, all blocks of information contained items that existed and items that do not exist but participants were only warned of the possibility of non-existent items in half of the blocks (Accountable condition vs. an Unaccountable condition where they were not warned that they might be claiming familiarity with something that does not exist). Increased MOFC activity was associated with the shift toward a more conservative standard for claiming knowledge in the Accountable condition. Each participant’s time series was extracted from the MOFC seed (5 mm radius sphere around group peak: −6, 58, −20 from the Accountable vs. Not Accountable contrast; Hughes and Beer, 2012b). Two PPI regressors were created: interaction of the time series of the MOFC seed with (i) the time series of the Accountable condition regressor and (ii) the time series of the Not Accountable condition regressor.

In Dataset 3 (Beer and Hughes, 2010), participants made the same social-comparative evaluations of their personality traits as in Dataset 1 but self-esteem threat was not manipulated. Instead, the breadth of personality traits were manipulated such that they could either be broadly construed (i.e., Broad condition: trait has a wide variety of behavioral manifestations such as “capable”) or more specifically construed (i.e., Specific condition: trait has few behavioral manifestations such as “talkative”). Reduced MOFC activity was associated with viewing the self as having more positive and fewer negative traits in comparison to the average peer (i.e., positively tinged evaluations of one’s personality). Each participant’s time series was extracted from the MOFC seed (5 mm radius sphere around group peak: −4, 46, −10 from the Specific vs. Broad contrast; Beer and Hughes, 2010). Two PPI regressors were created: interaction of the time series of the MOFC seed with (i) the time series of the Specific condition regressor and (ii) the time series of the Broad condition regressor.

After PPI regressors were created, all of the datasets were subjected to a subsequent fixed-effects analyses in the same manner as described earlier for the PPI analyses of the VACC seed. Specifically, the fixed-effects analyses to modeled condition of interest regressors (i.e., Dataset 1: Threat and No Threat conditions (Hughes and Beer, 2013; Dataset 2: Accountable and Unaccountable conditions (Hughes and Beer, 2012b; Dataset 3: Specific and Broad conditions (Beer and Hughes, 2010), a temporal filter of activity across the time course from the MOFC seed region, and the PPI regressors for conditions of interest. The PPI regressors were contrasted in a GLM. FEAT’s FLAME module (FMRIB’s Local Analysis of Mixed Effects; Smith et al., 2004) was used to preform mixed effects analyses for each dataset, which created group average maps for contrasts of interest (*p* < 0.005, uncorrected).

##### Results

**Figure 3:**
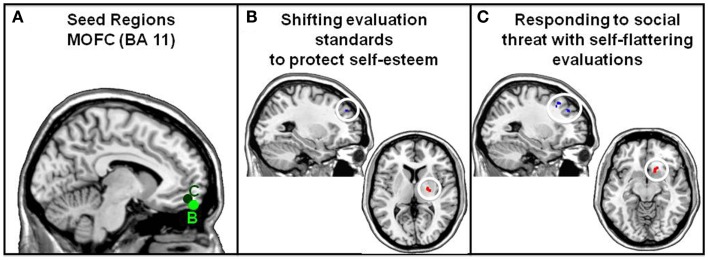
**PPI connectivity analyses for the MOFC seed associated with self-protection in the face of self-esteem threat**. **(A)** MOFC seed regions for connectivity analyses of previously published studies (5 mm radius spheres around group peak). Dark green seed: social comparisons while under Threat vs. No Threat contrast, group peak −12, 54, −14 (Dataset 1: Hughes and Beer, 2013). Light green seed: claims of knowledge while Accountable vs. Not Accountable contrast, group peak: −6, 58, −20 (Dataset 2: Hughes and Beer, 2012b). **(B)** When false claims of knowledge could be discovered, the MOFC seed associated with self-protection (i.e., less inflated claims) shows relatively more positive covariation with the right putamen (22, −10, 6) and less positive covariation with the left middle frontal gyrus (BA 9; −24, 52, 30). **(C)** When self-esteem was threatened, the MOFC seed associated with more self-flattering evaluations shows relatively more positive covariation with the right caudate (14, 16, −10) and less positive covariation with the middle frontal gyrus (BA 9).

PPI connectivity analyses (Friston et al., 1997) conducted on previously published results (Beer and Hughes, 2010; Hughes and Beer, 2012b, 2013) suggest that functional connectivity between MOFC, the striatum, and the middle frontal gyrus (BA 9) may support self-evaluations used to protect self-esteem in the face of threat (see Figure 3; Table 2). When self-esteem is at stake, the region of MOFC that is associated with self-evaluation shows relatively less positive covariation with middle frontal gyrus (BA 9) and relatively greater positive covariation with striatum. It is possible that functional connectivity between MOFC and striatal subregions reflects whether a shift to more conservative or liberal evaluation standards will be most rewarding in the face of self-esteem threat. For example, greater positive covariation between MOFC and caudate was found when liberal thresholds were advantageous for protecting self-esteem (Hughes and Beer, 2013) and between MOFC and putamen when conservative thresholds were advantageous for protecting self-esteem (Hughes and Beer, 2012b). Taken together, this research suggests that MOFC aids top-down influences on social cognition by adjusting evaluation standards as function of motivational state.

## Conclusions and Future Considerations for Research on the Role of MPFC, VACC, and MOFC in Self-Evaluation

While much progress has been made since the discovery in the early 2000s that MPFC is associated with self-evaluation, several hypotheses have been tested and eliminated. The new hypotheses described here will benefit from future research guided by a number of questions. For example, even though MPFC has received the bulk of attention, there are still many questions that remain. It would be extremely useful (and feasible) to conduct connectivity analyses on the large, existing body of studies that have measured MPFC modulation in relation to both self-evaluation and the evaluation of unknown others. One potential drawback of the “correction of self-projection” hypothesis for both VMPFC and DMPFC is that these regions are activated for evaluation of targets where correction of self-projection is unlikely (e.g., self and/or close others). If the functional connectivity of VMPFC and DMPFC is different during self-evaluation compared to evaluations of unknown others, those results would eliminate some concerns about the correction of self-projection hypothesis. Furthermore, more research is needed to decouple the affective vs. cognitive qualities shared by evaluation of the self and close others to more clearly delineate the role of VMPFC in social evaluation. If VMPFC is similarly modulated by the evaluation of another person where there is an emotional association with the self but no actual firsthand experience or basis for self-projection, then that would be strong evidence that VMPFC indexes the emotional aspect of self-relatedness when evaluating other people.

Additionally, more research is needed to clarify the possibility that VACC is involved in detecting opportunities that might fulfill expectations about self-evaluation. Does VACC mediate sensitivity to motivationally consistent information or positively valenced information when it is motivationally consistent? This question is important because psychological models show that motivation to see oneself in a positive light is not the only motivation that impacts self-evaluation. For example, the relation between valence and motivation becomes uncoupled when self-verification, another motivation known to influence self-evaluation, is activated. People often want to feel that their self-evaluations are correct and are vigilant for opportunities that have the potential to verify their current self-evaluations. In fact, this research finds that people with negative self-evaluations desire chances to confirm these negative self-evaluations (Swann et al., 1989). In this situation, the evaluation of negative traits (rather than positive traits) would have the potential to affirm motivated self-evaluation. If VACC mediates sensitivity to motivationally consistent information, then it should be modulated by opportunities to affirm a negative self-evaluation for people who are motivated to confirm a negative self-view. Furthermore, more research is needed to replicate and understand the psychological significance of the connectivity between VACC, MOFC and the other regions found in the PPI analyses.

Finally, more research is needed to replicate and elucidate the functional connectivity of MOFC in association with self-evaluations made in the presence and in the absence of self-esteem threat. While there is convergent evidence that more positive covariation with striatum and reduced covariation with middle frontal gyrus is associated with self-evaluation used to protect self-esteem, much less is understood about the significance of regions that covary with MOFC modulation associated with self-evaluations made in the absence of self-esteem threat.

In conclusion, a new framework is proposed to account for the contribution of MPFC, VACC, and MOFC to social cognition. MPFC is broadly implicated in self-evaluation but may be characterized by a ventral to dorsal division when evaluating others based on their intimacy. Certainty about evaluation may better characterize the contribution of DMPFC to social cognition than the correction of self-projection. The association between VMPFC and self-relatedness will be clearer once future research disentangles shared emotional and cognitive properties of evaluation of self and close others. Further, previous research has failed to take into account the fundamental role that motivation has in self-evaluations. As a result, the role of VACC and MOFC in social cognition has been obscured until recently. VACC may mediate bottom-up sensitivity to information based on its potential for helping us evaluate ourselves and others the way we want. MOFC may mediate top-down motivational influences on self-evaluation.

## Conflict of Interest Statement

The authors declare that the research was conducted in the absence of any commercial or financial relationships that could be construed as a potential conflict of interest.
